# Versatile Protein-A Coated Photoelectric Immunosensors with a Purple-Membrane Monolayer Transducer Fabricated by Affinity-Immobilization on a Graphene-Oxide Complexed Linker and by Shear Flow

**DOI:** 10.3390/s18124493

**Published:** 2018-12-18

**Authors:** Hsueh-Hsia Wu, Xin-Quan Liao, Xin-Ying Wu, Cheng-De Lin, Kai-Ru Jheng, Hong-Ren Chen, Yong-Yi Wang, Hsiu-Mei Chen

**Affiliations:** 1 School of Medical Laboratory Science and Biotechnology, College of Medical Science and Technology, Taipei Medical University, Taipei 11031, Taiwan; wuhh@tmu.edu.tw; 2 Department of Chemical Engineering, National Taiwan University of Science and Technology, Taipei 10607, Taiwan; letter605@hotmail.com (X.-Q.L.); ex8527@livemail.tw (X.-Y.W.); gn01657736@hotmail.com (C.-D.L.); D9906104@mail.ntust.edu.tw (K.-R.J.); kenneth991020@gmail.com (H.-R.C.); Bright4522@yahoo.com.tw (Y.-Y.W.)

**Keywords:** bacteriorhodopsin, graphene oxide, monolayer fabrication, photoelectric immunosensor, protein A, purple membrane, shear flow

## Abstract

Bacteriorhodopsin-embedded purple membranes (PM) have been demonstrated to be a sensitive photoelectric transducer for microbial detection. To efficiently prepare versatile BR-based immunosensors with protein A as antibody captures, a large, high-coverage, and uniformly oriented PM monolayer was fabricated on an electrode as an effective foundation for protein A conjugation through bis-NHS esters, by first affinity-coating biotinylated PM on an aminated surface using a complex of oxidized avidin and graphene oxide as the planar linker and then washing the coating with a shear flow. Three different polyclonal antibodies, each against *Escherichia coli*, *Lactobacillus acidophilus*, and *Streptococcus mutans*, respectively, were individually, effectively and readily adsorbed on the protein A coated electrodes, leading to selective and sensitive quantitative detection of their respective target cells in a single step without any labeling. A single-cell detection limit was achieved for the former two cells. AFM, photocurrent, and Raman analyses all displayed each fabricated layer as well as the captured bacteria, with AFM particularly revealing the formation of a massive continuous PM monolayer on aminated mica. The facile cell-membrane monolayer fabrication and membrane surface conjugation techniques disclosed in this study may be widely applied to the preparation of different biomembrane-based biosensors.

## 1. Introduction

In past decades, a great variety of immunosensors have been developed as alternatives to laborious and slow, classical culture-based methods for microorganism detection, including cantilever, conductometric, electrochemical, magnetic, optical, piezoelectric, and thermometric ones [1,2,3,4,5,6], as well as a novel photoelectric immunosensor that we recently proposed using a photosensitive bacteriorhodopsin (BR) as the transducer [7]. BR is the only protein component in the purple membrane (PM) of *Halobacterium salinarum*, natively existing in a trimeric form hexagonally packed as a highly compact and stable two-dimensional (2D) crystalline structure resistant to extreme pHs and temperatures [8]. Each BR monomer contains a retinal chromophore conjugated with Lys216, which isomerizes from the all-*trans* to the 13-*cis* conformation upon illumination, pumping protons unidirectionally from the cytoplasmic (CP) to the extracellular (EC) side of PM and consequently generating a photovoltaic force as well as an electrical impulse through the external circuit [7,8,9,10]. On the premise the bacterial cells captured close to PM block part of the incident light and thus reduce BR photocurrents, the previous BR-based immunosensor was constructed for the first time by anchoring cell-specific biotinylated antibodies through NeutrAvidin, a deglycosylated form of avidin, on the PM patches that had been unidirectionally immobilized on an electrode and then surface-conjugated with biotin on their exposed CP side [7]. The resulting immunosensor exhibited not only excellent selectivity and single-cell sensitivity but also a very wide dynamic range, allowing direct, label-free, and single-step detection of microorganisms without any sample labeling or other pretreatments.

*Staphylococcus aureus* protein A (SpA) has long been used as a universal tool to purify and directly immobilize antibodies in their immunologically active orientation due to its multivalent, specific, and strong binding affinity to the F_c_ regions of immunoglobulins G from several mammalian species [11,12]. For sensor applications, SpA-based electrochemical, optical, and piezoelectric immunosensors have been devised to detect microorganisms [2,3,13,14,15,16,17,18], with the nanoparticle-based electrochemical ones able to achieve low detection limits around the single-digit colony forming units (CFU)/mL [13,14]. Compared with the (strept)avidin-biotin interaction system, the immunosensor constructed based on the SpA-antibody interaction avoids previous antibody modification, which may result in alteration or hindrance of antigen-biding sites of the antibodies due to random biotinylation [19], as well as interference from biotin in the blood samples of patients who take high doses of biotin as a supplement or therapy [20,21].

This study aimed to develop another BR-based photoelectric immunosensor using SpA as an antibody capture to detect microorganisms. SpA was covalently attached on a PM-monolayer coated electrode through homobifunctional crosslinkers containing N-hydroxysuccinimide ester groups at either end (bis-NHS esters). PM monolayer fabrication was achieved by first affinity-immobilizing biotinylated PM, b-PM, on an aminated electrode via a planar complex linker constituted of graphene oxide (GO) and oxidized avidin (OA), and then washing the b-PM coated surface with a microfluidic shear flow. OA, which carries aldehyde groups, was the major component to bridge between b-PM and surface amines through its avidin-biotin affinity interaction and Schiff’s base linkage with each of them, respectively [7,22]. GO was employed as a solid surfactant to improve b-PM immobilization and surface smoothness [22]. Shear flow was applied with the intention to mobilize and increase the fluidity of the stacking b-PM patches to achieve high-coverage b-PM monolayer fabrication as well as effective subsequent SpA conjugation. The high versatility as well as high detection selectivity and sensitivity of this kind of SpA-based photoelectric immunosensors were demonstrated.

## 2. Materials and Methods

### 2.1. Materials

*Escherichia coli* K-12, *Bacillus subtilis* subsp. *spizizenii, Lactobacillus acidophilus*, and *Streptococcus mutans* were all obtained from the Bioresource Collection and Research Center (Hsinchu, Taiwan). b-PM and oxidized avidin (OA) were each prepared by modifying PM and avidin with EZ-link sulfo-NHS-LC-LC-biotin and sodium periodate, respectively, as previously described [22]. 3-Aminopropylphosphonic acid (APPA) and recombinant SpA were purchased from Fluorochem (Hadfield, UK) and ProSpec (Rotherham, UK), respectively. Avidin, bis(sulfosuccinimidyl) suberate (BS3), bis-NHS ester containing 5 polyethylene glycol (PEG) spacer units (Bis(NHS)PEG5), and SYTO 9 stain were all from Thermo Fisher Scientific (Waltham, MA, USA). The other two bis-NHS esters containing 2 and 11 PEG units, Bis(NHS)PEG2 and Bis(NHS)PEG11, respectively, were both from NANOCS (Boston, MA, USA). Rabbit polyclonal antibodies raised against *E. coli* all O and K antigens, keyhole limpet hemocyanin conjugated synthetic peptide derived from human *L. acidophilus* hypothetical protein, and *S. mutans* whole cells were obtained from ViroStat (Westbrook, ME, USA), Biorbyt (Cambridge, UK), and Abcam (Cambridge, UK) respectively. Graphene oxide (GO) powders and indium tin oxide (ITO) glass (sheet resistance: <15 Ω/sq) were from Graphene Supermarket (Calverton, NY, USA) and Fang Materials (New Taipei city, Taiwan), respectively.

### 2.2. Sensor Preparation and Characterization

ITO glass was first aminated with APPA as previously described [22], then drop-coated (coating diameter: 3 mm) with a mixture containing GO and OA in at least a 1:2 weight ratio, and b-PM in sequence, and finally washed with a laminar shear flow inside a microfluidic setup. The resulting b-PM chip was subsequently modified with 5 mM bis-NHS ester and 0.1 mg/mL SpA in sequence, blocked with 0.2 M glycine, and then coated with 25 μg/mL antibodies at pH 7.2–7.4 and 4 °C. The resulting antibody-coated b-PM chip was used to incubate with 10 mL of either bacterial samples or the blank cell-binding buffer (10 mM phosphate buffer, pH 7.4) and then washed with the same buffer. Photocurrents generated by b-PM chips were measured with a setup similar to that previously described [7]. As illustrated in Figure S1, the working b-PM coated electrode was placed on one side of a 1-cm path-length cuvette filled with 1 mL of the electrolyte (10 mM KCl, pH 8.5) and the counter electrode, a platinum bar, was situated at the farthest corner of the cuvette. Both the b-PM chip and the platinum bar were taped with copper foil tape and then wired to a homemade current amplifier using crocodile clips. The b-PM coated spot on the chip was front-illuminated with an aligned 80-mW green CW laser (beam diameter: 3 mm) and the generated photocurrent was real-time measured with the current amplifier and recorded with a digital oscilloscope. All the measurements were taken with the same geometrical arrangement of the illuminating laser beam, the counter electrode, and the b-PM chip, as well as with the same parameter settings of the measuring instruments. The total photocurrent density of a b-PM chip was defined as the difference between the maximum light-on ($I_{\mathrm{peak}-\mathrm{on}}$) and the minimum light-off ($I_{\mathrm{peak}-\mathrm{off}}$) photocurrent densities of the response to an on-and-off irradiation cycle. The total photocurrent density of an antibody-coated b-PM chip after being incubated with a cell sample was compared with the value of another control chip incubated with only the blank cell-binding buffer and the reduced percentage was defined as the photocurrent reduction level. Cell concentrations determined by the traditional plate count method were used as the standards. Atomic force microscopy (AFM) and Raman spectroscopy were performed as previously described [7], using freshly cleaved mica and ITO glass as the substrates, respectively. The coating procedures on mica were exactly the same as those on ITO. Fourier transform infrared (FTIR) spectra were recorded on a Nicolet 6700 spectrometer (Thermo Fisher Scientific, Waltham, MA, USA) by dropping a 200 μL of each sample solution on a CaF_2_ crystal plate.

## 3. Results and Discussion

### 3.1. Surface Fabrication and Characterization

**Figure 1:**
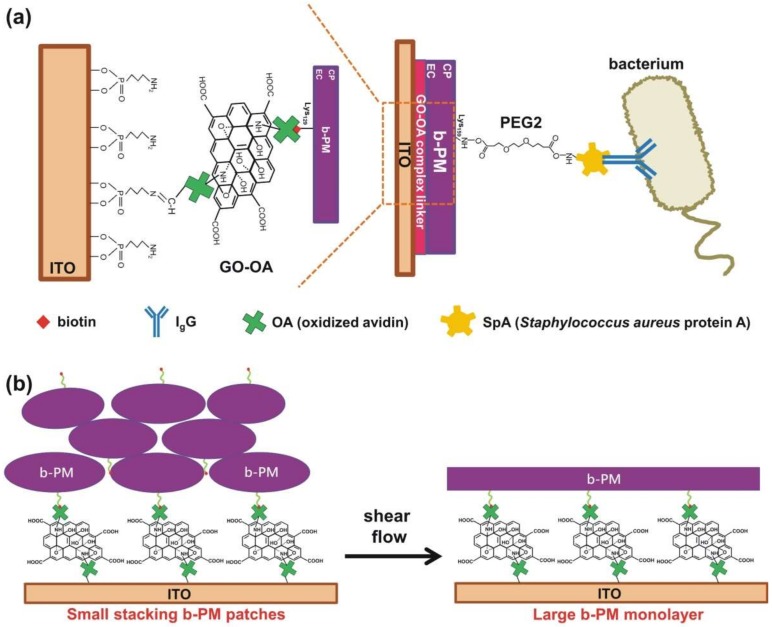
(**a**) Structural scheme of a SpA-based immunosensor prepared via Bis(NHS)PEG2 (right half) and structural details of linkages between b-PM and ITO (left half). IgG and PEG2 symbolize an antibody and Bis(NHS)PEG2, respectively. (**b**) Conceptual mechanism of the post-deposition washing procedure employed in the fabrication of a large b-PM monolayer on ITO.

Figure 1a depicts the structure of the proposed SpA-based photoelectric immunosensor, where b-PM, a PM conjugated with biotin at its EC side, was unidirectionally deposited on ITO through biotin-avidin interaction (Figure 1a, left half) and used as the sensor transducer. Such a unidirectional PM fabrication was proposed to achieve the greatest photocurrent production of the pristine transducer because BR molecules in this orientation, with their EC side facing electrodes, reportedly produced much higher photocurrents than the ones in the other orientation [23]. In addition, the layer-by-layer sensor structure shown in Figure 1a not only ensured a reliable and reproducible fabrication scheme of the sensor, which combined self-assembled monolayer technique, covalent linking, and avidin-biotin affinity adsorption, but also provided a robust and stable attachment of the b-PM transducer layer on the electrode as well as of the recognition molecules on the transducer. In the fabrication, a robust and amine-terminated self-assembled monolayer, which facilitated the subsequent conjugation of biomolecules and avoided the surface denaturation of proteins, was first formed on ITO electrodes through the phosphonate headgroup of APPA. The planar double-sided GO-OA complex linker was then attached onto the aminated substrate through the Schiff’s base linkage between the terminal amines of APPA and the aldehydes of oxidized sugars on avidin. Finally, b-PM was affinity-captured on the linker. For covalent SpA conjugation on the exposed surface of the b-PM transducer, a homobifunctional amine-reactive reagent, bis-NHS esters, was used as the linker.

Different from the previous b-PM fabrication scheme employed in our first BR-based biosensor study, where only free OA was used as the linker for b-PM affinity immobilization [7], we proposed a modified, yet more effective process in the current study to eliminate the potential inter-crosslinking between counter-oriented stacking b-PM patches by homobifunctional bis-NHS esters during the subsequent SpA conjugation step. The process involved first utilizing a simple mixture of GO and OA as a planar complex linker for b-PM coating and then washing the surface of the b-PM coated substrate with a microfluidic shear flow. The addition of GO into the linker was proposed according to our previous finding that the utilization of a complex linker constituted of biotinylated GO and OA yielded a b-PM coating morphology with less stacking, more compact interior, and a smoother surface than the morphology obtained when only free OA was used as the linker [22]. The positive effect was attributed to the 2D and surfactant characteristics of GO sheets, which not only inhibited b-PM and OA aggregation but also provided a flat support for b-PM attachment. Nevertheless, that study also showed biotinylated GO sheets tended to flocculate and precipitate due to their reduced edge-to-edge electrostatic repulsion, and the surfactant-like property of GO as well as the increased hydrophobicity of biotinylated GO caused partial disintegration of the immobilized b-PM patches. Therefore, the current study directly used hydrophilic unmodified GO as an adjunct planar linker to avoid the expensive, laborious, and meticulous preparation of biotinylated GO, as well as the problems of GO self-aggregation and b-PM disintegration. Due to the intrinsic fluid nature of cellular membranes, the post-deposition washing procedure was subsequently introduced to mobilize and disintegrate the upper stacking b-PM layers, with the intention to fill up the initially uncovered complex linkers with those dislocated cracked patches so a mostly uniformly oriented, nearly laterally continuous and full-coverage b-PM monolayer could be formed, as illustrated in Figure 1b. Moreover, it was postulated that on the resulting large unidirectional b-PM monolayer, further surface functionalization and molecular attachment could be optimally achieved, enabling one end of a bis-NHS ester crosslinker to conjugate with Lys159 on the exposed CP side of the immobilized b-PM monolayer, thus leaving the other end of the crosslinker available for SpA to attach through its abundant Lys residues [24], as depicted in the right half of Figure 1a

**Figure 2:**
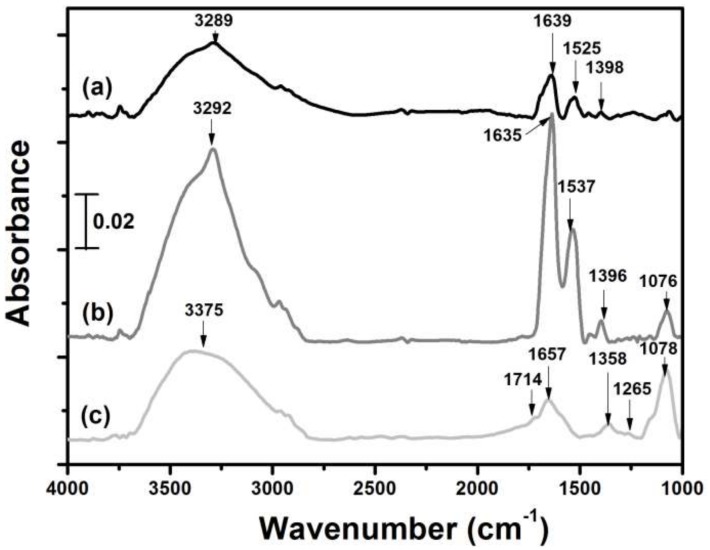
FTIR spectra of (**a**) pure OA, (**b**) a mixture of GO and OA (1:5 weight ratio), and (**c**) pure GO. The peak assignments of all the spectra are summarized in Table S1.

Figure 2 and Table S1 show the FTIR spectra and peak assignments of pure GO, pure OA, and a mixture of GO and OA. The GO spectrum displayed most of the previously reported characteristic absorption bands [25]. Pure OA exhibited a spectrum similar to that of native avidin [26], in accordance with the previous finding that both avidin and OA had similar secondary structures [27]. The 1396–1398 cm^−1^ signal of pure OA, which was absent in pure GO and could be attributed to C-N stretching [28], was initially small, but became stronger with the addition of GO, suggesting covalent linkages between the amines of OA and epoxides of GO basal planes [29]. The binding was further confirmed by AFM analysis of the planar GO-OA complex linker, showing the attachment of globular OA on either side of a GO sheet (Figure S2b). Strong hydrogen binding between GO and OA was also possible, as evidenced by significant upsurge of the broad near-Gaussian band at 3288–3375 cm^−1^ of the mixture in Figure 2 [30] as well as the massive stacking aggregates appearing in Figure S2a. Interestingly, both of the Amide I and Amide II signals of pure OA at 1635–1639 and 1525–1537 cm^−1^, respectively, which were also absent in GO, were augmented following GO addition, implying GO binding enhanced the secondary structure of OA, especially its major antiparallel β-sheets, because those two signals are considered to have strong correlation with the protein secondary structure [31]. Moreover, the ratio of Amide II/Amide I signals of the complex mixture was nearly similar to that of pure OA (0.485 vs. 0.455), implying most of the native structure of OA had been preserved following GO binding according to the previous finding that surface-denatured avidin had a much higher ratio than the native one [32].

**Figure 3:**
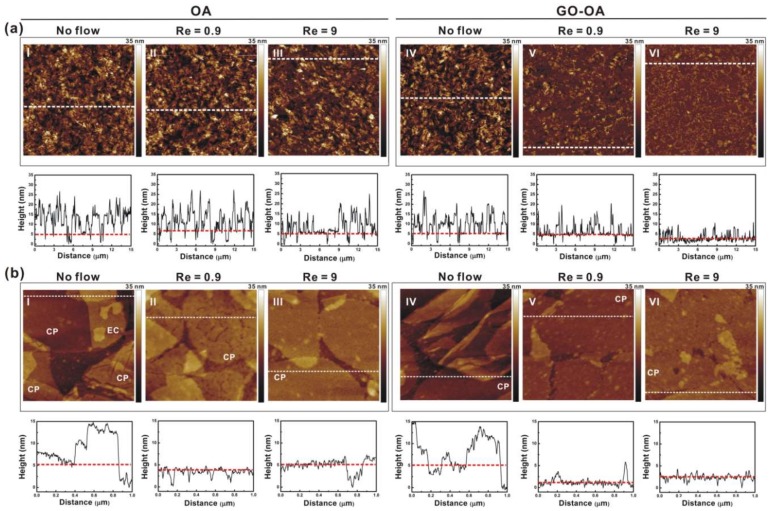
(Upper) AFM topographic images and (lower) sectional profiles of b-PM layers fabricated on (I–III) pure OA-coated and (IV–VI) GO-OA complex liker-coated mica. b-PM coated mica was subjected to AFM analysis (I,IV) without and (II,III,V,VI) with post-deposition washing with a (II,V) low and (III,VI) high microfluidic shear flow. Scan size: (**a**) 15 μm and (**b**) 1 μm. Sectional profiles were analyzed along the white dotted lines on the images, with the bottommost b-PM monolayers indicated by red dotted lines. EC and CP denote the exposed side of each b-PM patch.

The geometric arrangement of OA on both basal planes of a planar GO sheet benefited not only immobilization of the planar complex linker on the surface but also b-PM attachment. As depicted in the left half of Figure 1a, the planar GO-OA complex linker could be affixed on an aminated substrate through Schiff’s base linkages between the surface amines and the aldehydes of OA attaching on its lower basal plane, allowing b-PM to be subsequently affinity-captured in a stretching 2D conformation by OA attaching on its upper basal plane. As revealed by the AFM analysis on mica in Figure 3(a-I,a-IV,b-I,b-IV), on both pure OA-coated and complex liker-coated substrates, b-PM patches were initially deposited in their intact form, distributed unevenly, and stacked considerably in about 5–6 and 4–5 layers, respectively. In addition, tiny zones of uncovered linkers were observed scattering all over both primitive b-PM coated substrates. Such a dense b-PM stacking was possibly due to the hydrophilicity of the linker as well as the ionic interaction between counter-oriented b-PM patches, as indicated in Figure 3(b-I). However, the subsequent post-deposition washing procedure had distinctly different effects on these two different b-PM coated surfaces. After the washing, there remained 2–3 b-PM layers stacking on the pure OA-coated substrate (Figure 3(a-II,a-III)), with most of the b-PM patches staying in their initial intact, separate, crack-structured, and slightly elliptical forms (Figure 3(b-II,b-III)). The washing with a stronger shear flow (Reynolds number, Re = 9) only resulted in improving the interior compactness and surface smoothness of each b-PM patch (Figure 3(b-III)). 

On the other hand, profound reduction of b-PM stacking by microfluidic washing was achieved on the b-PM surface prepared via the linker complexed with GO. When simply washing with a slow shear flow (Re = 0.9), most of the upper stacking b-PM layers were effectively removed, exposing a great majority of the bottom layer composed of separate adjacent monolayer patches (Figure 3(a-V)). The surface area covered only by single b-PM monolayers increased from 21% to 91% after the slow washing procedure. Further close-up topographic analysis of those b-PM monolayers revealed most of them no longer looked slightly elliptical, and had fused with neighboring monolayers and hence become larger in size (Figure 3(b-V)), suggesting the occurrence of dynamic transitions and redistribution of b-PM monolayers during the washing step, similar to our previous observations [7,10]. Examining the interior membrane structures in the close-up images of those flow-treated b-PM surfaces revealed the initial cracked structure in the bottom layer in Figure 3(b-I,b-II,b-IV), which was identified as the CP side of PM [33], no longer appeared in Figure 3(b-V), indicating reorganization of b-PM monolayers during the flow-assisted fusion process. The fusion was even more effective with a stronger shear flow (Re = 9), making almost all the bottom b-PM monolayers combine together to form a very large, nearly laterally continuous single monolayer covering on the substrate (Figure 3(a-VI,b-VI)). The tiny extra layers sparsely topping the large continuous foundation layer were possibly the debris of fragmented membranes.

**Figure 4:**
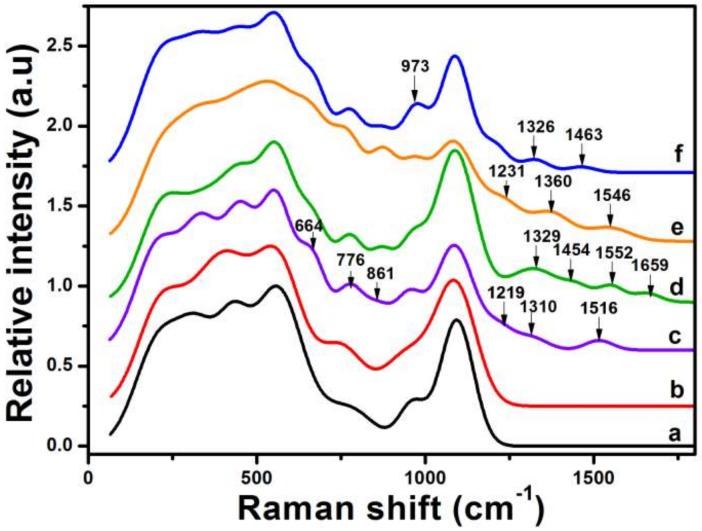
Normalized Raman spectra of ITO electrodes fabricated with (**a**) APPA, (**b**) GO-OA complex liker, (**c**) b-PM, (**d**) SpA, (**e**) anti-*E. coli* antibodies, and (**f**) *E. coli* K-12 cells at the top. The b-PM surface prepared via the complex linker and subsequently washed with a microfluidic shear flow was used for the analysis as well as in the following coatings. Bis(NHS)PEG2 was used for SpA conjugation.

Raman analysis of the layer-by-layer fabricated ITO substrates confirmed not only SpA conjugation on b-PM but also subsequent antibody and microbe binding. Figure 4 shows the normalized Raman spectra of ITO electrodes fabricated with different topmost layers, with band identification and assignment shown in Figure S3 and Table S2, respectively, according to the previously referred to studies. Possibly due to scanty coating amounts, the characteristic bands of neither GO at around at 1355 and 1600 cm^−1^ [34] nor OA above 1200 cm^−1^ [7] were observed in the spectra of the substrate topped with the GO-OA complex linker. Nevertheless, subsequent b-PM coating followed by microfluidic washing resulted in the appearance of additional bands at 664, 776, 861, 1219, 1310, and 1516 cm^−1^ caused by the amide III, C=C stretches, C-C-H in-plane bends, C-S stretches, Trp, and Tyr of BR. Further SpA conjugation on such a b-PM surface through Bis(NHS)PEG2, a bis-NHS ester with 2 PEG spacer units, caused not only the disappearance of the 1310 and 1516 cm^−1^ bands of retinal but also the addition of the 1329, 1454, 1552, and 1659 cm^−1^ bands assigned to amide III, CH_2_ deformation, Trp, and amide I of SpA. Subsequent anti-*Escherichia coli* antibody immobilization was evidenced by the bands appearing at 1231 and 1360 cm^−1^ attributed to amide III and Trp, respectively. Finally, the intensified band at 973 cm^−1^ as well as the weak bands at 1326 and 1463 cm^−1^ in Figure 4f were assigned to the captured *E. coli* cells.

**Figure 5:**
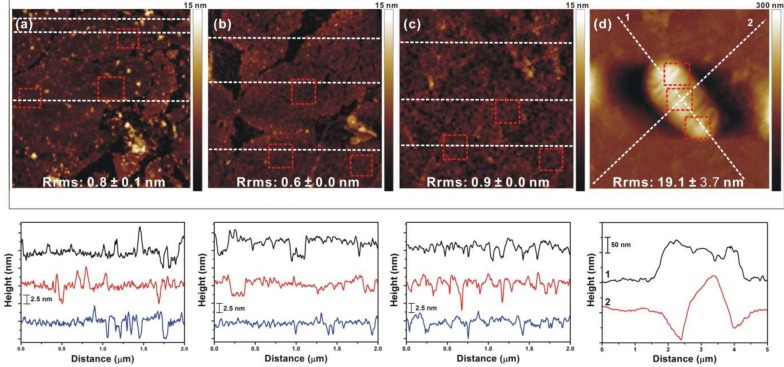
(Upper) AFM topographic images and (lower) sectional profiles of different layer-by-layer fabricated mica. The topmost layer in each image was (**a**) b-PM, (**b**) SpA, (**c**) anti-*E. coli* antibodies, and (**d**) *E. coli* K-12 cells. Scan size: (**a**–**c**) 2 μm, (**d**) 5 μm. The b-PM surface prepared via the complex linker and subsequently washed with a microfluidic shear flow was used for the analysis as well as in the following coatings. Bis(NHS)PEG2 was used for SpA conjugation. Red dotted boxes in the images indicate the sectioned areas within each topmost layer for Rrms estimation. Sectional profiles were analyzed along the white dotted lines on the images.

In addition, AFM was used to analyze layer-by-layer fabricated mica, with the b-PM layer deposited via the complex linker and subsequently washed with a microfluidic shear flow. As shown in Figure 5, the topographic images of different topmost layers were distinctly dissimilar to one another and had different average root mean square roughness (Rrms), indicating successive material coatings. Similar to what was observed in Figure 3(b-V), most of the foundation b-PM layer appeared as a large, continuous, 5.0 ± 1.0 nm-thick, and densely packed single monolayer (Figure 5a). The topmost SpA layer shown in Figure 5b was 4.9 ± 1.2 nm thick, suggesting most SpA molecules were attached in a “brush-like” protrusion structure with their “brush-like” portion first extended into the solution and then compressed to the bottom immobilized portion in a dry state [35,36]. Interestingly, the surface of the SpA layer was no longer as continuous and as large as that of the b-PM layer, but instead appeared as a discontinuous terrain composed of contiguous smaller patches with a cracked structure and slightly elliptical shape, which was similar to the initial conformation of the CP side of unwashed b-PM patches immobilized at the bottom layer in Figure 3(b-IV). Since there were only 2 lysines exposed on the PM surface (PDB: 4Y9H), Lys129 and Lys159 on the EC and CP sides, respectively, and the NHS-ester biotinylation reagent used to prepare b-PM has a conjugation preference on Lys129 [37], it was likely Lys159 was the only remaining site on b-PM for SpA to conjugate with through Bis(NHS)PEG2. Therefore, SpA possibly attached only on the immobilized b-PM patches with their CP sides exposed, resulting in the crack and patch morphology obtained in Figure 5b. Antibody coating was evident in Figure 5c, not only by the increased thickness of the coating layer, which was 6.3 ± 1.8 nm and similar to the previous finding [38], but also by the appearance of a loose and thread-like structure resembling a closely packed layer of Y-shaped immunoglobulins. Finally, *E. coli* binding on the antibody-coated surface was confirmed by the rod-shaped cell images in Figure 5d.

### 3.2. Photocurrent Response and Quantitative Cell Detection

**Figure 6:**
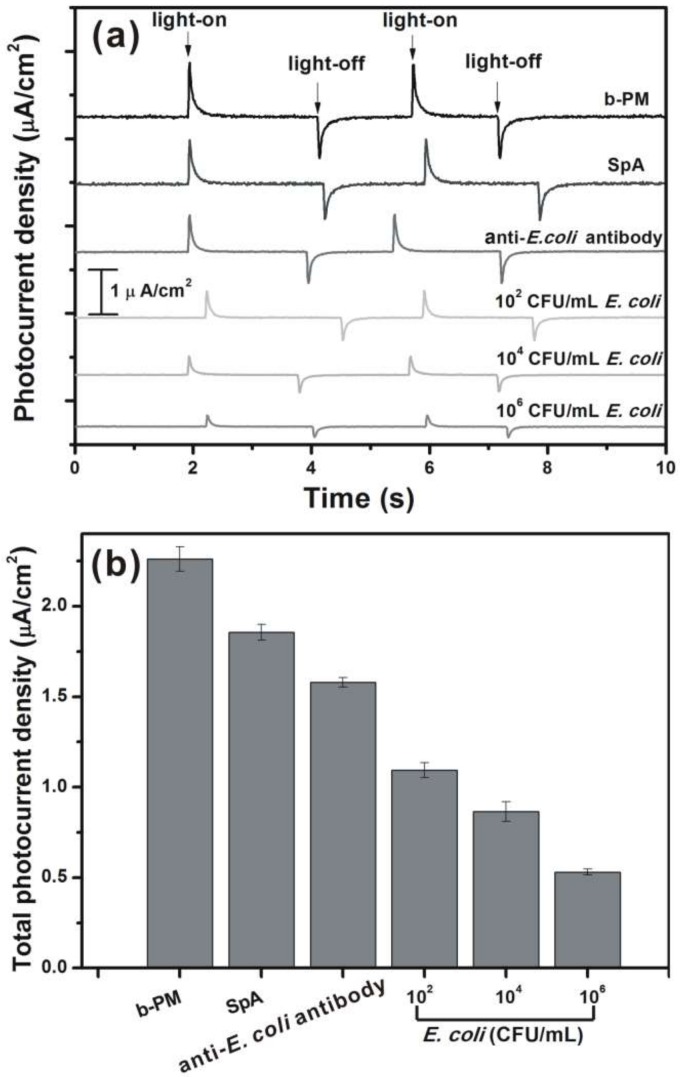
(**a**) Typical photocurrent responses and (**b**) total photocurrent densities of the chips fabricated with different topmost layers. b-PM chips prepared via the complex linker and washed with a microfluidic flow were used for the subsequent coatings. Bis(NHS)PEG2 was used for SpA conjugation. Light-on and light-off in (**a**) indicate the responses of a chip when the irradiation was performed and interrupted, respectively. All of the data shown in (**b**) represent the average of three chips of a single kind with one standard deviation.

Figure 6 shows the typical photocurrent responses and the total photocurrent densities of different ITO electrodes, each respectively fabricated with b-PM, SpA, anti-*E. coli* antibodies, and *E. coli* K-12 cells at the top. The pristine, unwashed b-PM chips prepared via the GO-OA complex liker generated 120 ± 3% higher total photocurrent densities (responses not shown) than the presented ones that had been washed with a microfluidic shear flow, suggesting the bottommost b-PM layers closely attached to ITO contributed much more than the upper stacking layers in terms of photocurrent generation (83% vs. 17%). Similar photoelectric activities and photocurrent reduction behavior in post-deposition washing were also observed with the other b-PM chips prepared using only free OA as the liker. However, because b-PM stacking remained significant on this kind of washed b-PM chips, as shown in Figure 3(a-II,a-III), only the washed b-PM chips prepared via the GO-OA complex liker were used in the subsequent immunosensor study. Successive coatings on top of the washed b-PM chips prepared via the GO-OA complex liker resulted in a gradual decline in photocurrent generation, as shown in Figure 6. In addition, the detection with the antibody-coated chips prepared with prior microfluidic washing resulted in decreased total photocurrent densities with increasing *E. coli* concentrations, indicating successful cell binding. As compared in Table S3, the SpA-coated chips we initially obtained without prior microfluidic washing failed to exhibit significant photocurrent reduction in both subsequent antibody binding and *E. coli* detection steps. In addition, the relative standard deviations (RSDs) of the photocurrents of those non pre-washed chips were considerably larger than those of the pre-washed ones. Therefore, the simple addition of the novel post-deposition washing procedure in chip fabrication markedly improved the efficiency, repeatability, and reproducibility of SpA and antibody immobilization as well as cell binding. Further, four different bis-NHS ester crosslinkers, BS3, Bis(NHS)PEG2, Bis(NHS)PEG5, and Bis(NHS)PEG11, containing 0, 2, 5, and 11 PEG spacer units, respectively, were used to conjugate SpA and then to adsorb anti-*E. coli* antibodies onto the washed b-PM chips.

All four resulting immunosensing chips exhibited considerable and similar photocurrent reductions (45.9%, 47.8%, 51.2%, and 43.8%, respectively) in the detection of a 10^5^ CFU/mL *E. coli* K-12 culture. Those results supported our previous postulation that the nearly continuous and unidirectional b-PM monolayers prepared with the aid of shear flow as well as GO complexed with OA provided a potent and robust foundation for effective SpA conjugation through the versatile bis-NHS ester crosslinkers.

**Figure 7:**
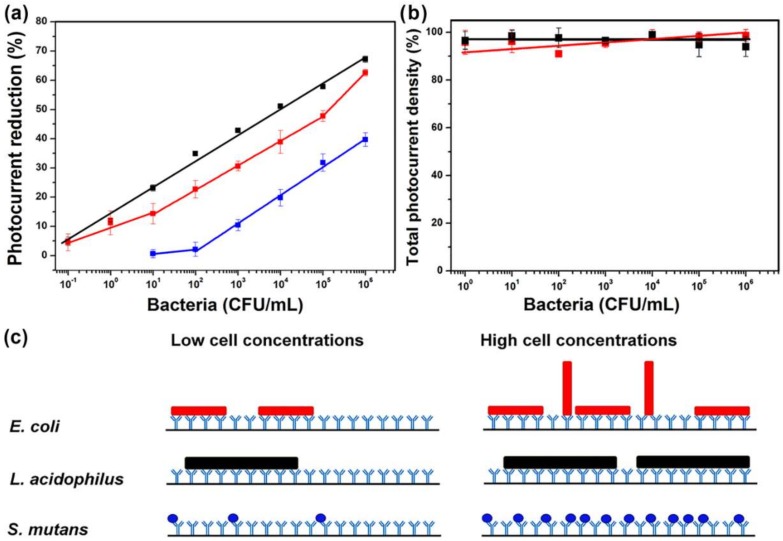
(**a**) Calibration curves of (red) *E. coli*, (black) *L. acidophilus*, and (blue) *S. mutans* immunosensing chips on the detection of *E. coli* K-12, *L. acidophilus*, and *S. mutans* cultures, respectively. (**b**) Relative total photocurrent densities of *E. coli* immunosensing chips on the detection of (red) *B. subtilis* and (black) *L. acidophilus*. The average of the total photocurrent densities generated by the fresh *E. coli* sensing chips incubated with only the blank cell-binding buffer was taken as 100% in (**b**). b-PM chips prepared via the complex linker and washed with a microfluidic flow were used to prepare all kinds of immunosensing chips. Bis(NHS)PEG2 was used for SpA conjugation. All of the data represent the average of three chips of a single kind with one standard deviation. (**c**) Schematic illustrating possible arrangements of different bacteria adsorbed on their respective immunosensing chips at low and high cell concentrations. For simplicity, the chips were represented with only their topmost antibody layers.

*E. coli*, *L. acidophilus*, and *S. mutans* immunosensing chips were each prepared with the as-prepared SpA-coated electrodes and their calibration curves are shown in Figure 7a. Each kind of cells captured on their respective antibody-coated chips were simultaneously examined with fluorescence microscopy, all indicating that the stained cell numbers increased with the sample cell concentrations (Figure S4), which agreed with the monotonically increasing trend of the calibration curves obtained by photocurrent measurements. Therefore, the photocurrent reduction levels of the sensing chips upon cell binding can be taken as a ready-to-read parameter to quantitatively determine bacterial concentrations without tedious microscopic cell counting. As observed in Figure 7b, the *E. coli* immunosensor exhibited hardly any significant decline in photocurrent generation after the incubation with either *B. subtilis* or *L. acidophilus* at different cell concentrations, suggesting that both Gram-positive cells were scarcely recognized and captured by the sensor. Compared with other SpA-based immunosensors for microbial detection, the current photoelectric one has not only both advantages of direct detection and label-free assay, which may benefit its future commercial applications, but also the lowest detection limit and the widest dynamic range (Table S4). Even in comparison with other direct and label-free immunosensors [5,39], whose detection principles were based on electrochemical impedance spectroscopy and single-walled carbon nanotube-based multi-junction, respectively, a lower detection limit and a wider dynamic range can still be obtained with the current invention.

Figure 7a also shows that both *E. coli* and *L. acidophilus* sensing chips exhibited a wide 7-log detection range and a 5% photocurrent reduction at the low detection limit of 1 CFU/10 mL, which was equivalent to one single cell. In the future, the sensor sensitivity at the lowest detection limit could be improved if the immobilization efficiency of antibodies can be further increased by optimizing the reaction conditions for SpA and antibody conjugations. Compared with the *E. coli* sensing chip, the *L. acidophilus* one had not only a better sensitivity below 10^2^ CFU/mL (Table S5), which is defined as the slope of the calibration curve, but also a larger photocurrent reduction at 10^6^ CFU/mL. On the other hand, the *S. mutans* sensing chip had a detection limit of 10^2^ CFU/mL and hence exhibited only a 40% photocurrent reduction at 10^6^ CFU/mL. The sensitivities of these three chips were similar to one another in the range of 10^2^–10^5^ CFU/mL (Table S5). Both *E. coli* and *L. acidophilus* cells have rod shapes, with 0.5 μm × 2 μm and 0.5–1.2 μm × 1–10 μm in dimensions, respectively, while *S. mutans* cells are smaller spherical cells, being only 0.5–0.75 μm in diameter. Therefore, the above-mentioned discrepancies suggest the dimensions of the target cells affected the detection limits of the as-prepared photoelectric immunosensors, as well as the detection sensitivity at low cell concentrations (<100 CFU/mL). At higher cell concentrations, the sensitivity of the *E. coli* sensing chip was gradually increased, while the sensitivities of both *L. acidophilus* and *S. mutans* chips were kept almost unchanged. The finding implied there might be different adsorption modes for *E. coli* at different cell concentrations, as postulated in Figure 7c. At low concentrations, *E. coli* cells were adsorbed mainly with their long sides parallel to the substrate surface. When there were more cells captured at higher concentrations, some of them might be adsorbed vertically due to space limitation as well as the fact the antibody-recognized capsular K and somatic O antigens are distributed all around the whole *E. coli* cells. The fraction of vertically adsorbed cells could become larger when the cell concentration was further increased, resulting in a surge of sensitivity in the range of 10^5^–10^6^ CFU/mL (Table S5). Nevertheless, both *L. acidophilus* and *S. mutans* cells were adsorbed in only one orientation on their antibody surfaces due to their large aspect ratio and spherical shape, respectively (Figure 7c). Vertical adsorption of *L. acidophilus* cells on their short sides could be hindered because of their overly long length, resulting in the observation of only one slope in the calibration curve.

As proposed in the previous study [7], the light-on ($I_{\mathrm{light}-\mathrm{on}}$) and light-off ($I_{\mathrm{light}-\mathrm{off}}$) transient photocurrent density signals generated by a b-PM coated chip could be simulated by the following two equations, respectively:

(1) $$I_{\mathrm{light}-\mathrm{on}}\;=\;\frac{E_{0}}{R_{p,\mathrm{on}}}\;\exp\left(-\frac{t}{\tau_{p,\mathrm{on}}}\right),\;\;\;\tau_{p,\mathrm{on}}{\;=\;R}_{p,\mathrm{on}}{\;C}_{p}$$

and:

(2) $$I_{\mathrm{light}-\mathrm{off}}\;=\;-\frac{E_{0}}{R_{p,\mathrm{off}}}\;\exp\left(-\frac{t}{\tau_{p,\mathrm{off}}}\right),\;\;\tau_{p,\mathrm{off}}{\;=\;R}_{p,\mathrm{off}}{\;C}_{p}$$

where $E_{0}$ is the constant value of $E_{p}$, the photoemf of the photocurrent source, produced during the continuous illumination; $R_{p,\mathrm{on}}$ and $R_{p,\mathrm{off}}$ are the internal resistances of $E_{p}$ encountered by light-on and light-off transient photocurrents, respectively; $C_{p}$ is the chemical capacitance passed through by transient photocurrents, which is considered the same for both opposite signals of the same irradiation cycle because it was generated by the overall illuminated b-PM layer. $\tau_{p,\mathrm{on}}$ and $\tau_{p,\mathrm{off}}$ are the decay-time constants of the light-on and light-off signals, respectively. Therefore, $I_{\mathrm{peak}-\mathrm{on}}{\;=E}_{0}/R_{p,\mathrm{on}}$, $I_{\mathrm{peak}-\mathrm{off}}=-E_{0}/R_{p,\mathrm{off}}$, and the total photocurrent density= ($I_{\mathrm{peak}-\mathrm{on}}-{\;I}_{\mathrm{peak}-\mathrm{off}}$). Accordingly, $\left|I_{\mathrm{peak}-\mathrm{on}}\right|/\left|I_{\mathrm{peak}-\mathrm{off}}\right|{=R}_{p,\mathrm{off}}/R_{p,\mathrm{on}}$, that is, the peak ratio of the light-on and light-off signals in each photocurrent response equals to the inverse ratio between the resistances encountered by the light-on and light-off photocurrents, respectively.

To procure all the parameters in the above equations to reveal the mechanisms of the photocurrent response and the photocurrent reduction of the current sensors, further studies are required in the future by employing sophisticated instruments to measure the capacitance as well as to investigate the light absorption and scattering behaviors of each material-coated b-PM chip. Nevertheless, the major factor causing photocurrent reduction of the immunosensor on cell binding may be indirectly inferred as below. First, in the previous investigation on the effect of illumination orientation on the photocurrent behaviors of different b-PM coated chip, we have already found that the front-illuminated chips always exhibited a much more significant photocurrent reduction upon layer-by-layer material coating than the back-illuminated chips, and hence suggested that the light-shielding effects of the materials deposited on b-PM was the major factor for photocurrent reduction [7]. In addition, the light-on and light-off transient signals responding to the onset and the cessation of illumination have been attributed to the charging and discharging processes of the chemical capacitance, respectively [40]. The charging was powered by unidirectional transportation of the pumped proton from the CP to the EC side of PM by illuminated BR. As shown in Table S6, all the calculated peak ratios are greater than 1, implying that all kinds of b-PM coated electrodes prepared in this study encountered a larger resistance in the discharging process than in the charging process. If $R_{p,\mathrm{on}}$ was further assumed nearly the same for all the kinds of b-PM coated chips, which might be possible based on the fact that all the chips herein were prepared with the same composite-spacer structure between the ITO electrode and the EC side of b-PM, the variation trend of the ratios in Table S6 may be simply considered as the variation trend of $R_{p,\mathrm{off}}$. 

For each kind of immunosensors, cell binding at different concentrations hardly caused significant variations of the ratios; therefore, $R_{p,\mathrm{off}}$ could be also considered nearly the same for all the immunosensors bound with different amounts of target cells. Therefore, the dramatic decays of both $\left|I_{\mathrm{peak}-\mathrm{on}}\right|$ and $\left|I_{\mathrm{peak}-\mathrm{off}}\right|$ caused by cell attachment might be attributed entirely to $E_{0}$ reduction, i.e., the decrease of photoemf, which was resulted from the decline of the illumination intensity on b-PM after cell binding.

To carry out the stability study, the as-prepared *L. acidophilus* immunosensing chips were stored in a 10 mM phosphate buffer containing 150 mM NaCl (pH 7.4) at 4 °C for 2–8 days and then used to detect a 10^4^ CFU/mL *L. acidophilus* culture. None of the chips lost their either photocurrent generation ability or cell binding affinity (Figure S5), yielding almost the same total photocurrent densities as well as photocurrent reduction levels as the fresh chips did. Finally, we tested different diluted commercial drinkable yogurts with our *L. acidophilus* sensing chips as well as with the commonly used enzyme-linked immunosorbent assay (ELISA) method. 

**Figure 8:**
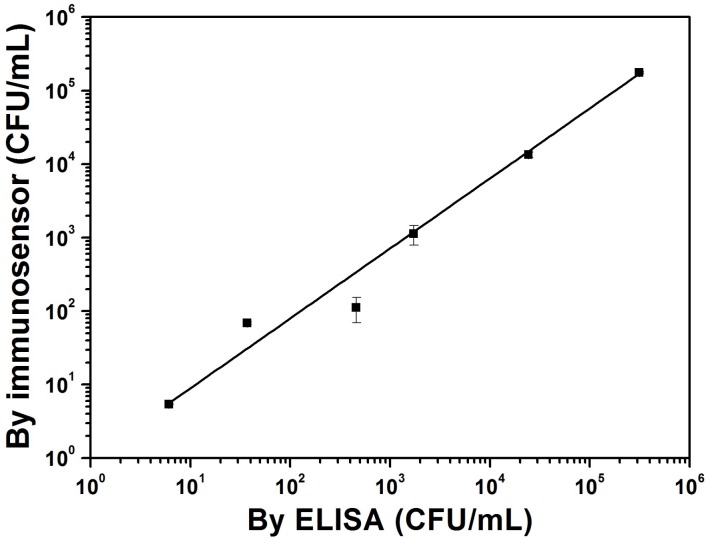
Comparison between the *L. acidophilus* cell concentrations estimated by the current *L. acidophilus* immunosensor and by the ELISA method for different diluted commercial drinkable yogurts. Drinkable yogurts from three different commercial brands were used to prepare the diluted samples in the cell binding buffer at low (1–10^2^ CFU/mL), medium (10^2^–10^4^ CFU/mL), and high (10^4^–10^6^ CFU/mL) cell concentrations, respectively. The ELISA test was performed on a Falcon 96-well microplate (Corning, Corning, NY, USA) that had been directly coated with anti-*L. acidophilus* antibodies and blocking glycine in sequence. After the adsorption with either different diluted pure *L. acidophilus* cell cultures, which were used as the standards for the calibration curve, or the diluted drinkable yogurt samples, the captured cells were first identified with the primary anti-*L. acidophilus* antibodies and then fluorescently labelled with goat anti-rabbit IgG H&L conjugated with Alexa Fluor^®^ 488 (Abcam, Cambridge, UK). The ELISA signals were analyzed with a Synergy H1 Multi-Mode microplate reader (BioTek, Winooski, VT, USA). No fluorescent signal was observed for the control experiment without adding cells in the tested solution. The immunosensor data represent the average of three chips with one standard deviation. The line was obtained by fitting the data with a power-law equation.

The results shown in Figure 8 indicate that the *L. acidophilus* cell concentrations estimated by both methods were well correlated with each (power law exponent = 0.95 ± 0.01; adjusted R-square = 0.899). Both sets of the estimated cell concentrations were of the same order of magnitude, with the values obtained by the ELISA method slightly larger than the ones by the immunosensor possibly because of greater unspecific adsorption of other co-cultured yogurt cells on the polystyrene ELISA-plate surface. Compared with the ELISA method, which requires two additional steps to fluorescently label the captured cells, the current microbial detection method using our BR-based photoelectric immunosensor is more cost-effective, direct, and rapid.

## 4. Conclusions

A novel fabrication scheme, which combined the surfactant-like effect of GO as well as the mobilization and segregation effects of shear flow, was proposed to form a large, mostly uniformly oriented, and nearly laterally continuous b-PM monolayer on an aminated substrate to provide a potent and robust foundation for effective SpA immobilization. The added GO sheet not only acted as an adjunct planar linker to make the pristine globular OA linkers dispersedly conjugated on its both sides but also provided a flat support for b-PM attachment. Versatile immunosensors were readily prepared to perform direct, label-free, selective, sensitive, and single-step quantitative detection of microorganisms. In addition, the AFM-confirmed structure of the sensing chip, where a monolayer of antibodies was fabricated in parallel with the underneath monolayer of light-sensing BR molecules, enabled us to demonstrate the size effect of the target cells on the sensor sensitivity, supporting the postulated light-blocking detection principle of the BR-based immunosensor. The same fabrication and conjugation principles can be further applied to prepare other BR-based biosensors with peptides, proteins, or amine-carrying functional materials as the recognition elements. In addition, the test on field samples suggested that our current microbial detection method was not only comparable to the commonly used ELISA technique, but also more cost-effective, direct, and rapid.

## Supplementary Materials

The following are available online at http://www.mdpi.com/1424-8220/18/12/4493/s1, Figure S1: Equipment setup for photocurrent measurement, Figure S2: AFM (a, b) topographic images and (c, d) sectional profiles of a GO-OA complex linker deposited on aminated mica, Figure S3: Deconvoluted Raman spectra of ITO electrodes fabricated with (a) APPA, (b) GO-OA complex liker, (c) b-PM, (d) SpA, (e) anti-*E. coli* antibodies, and (f) *E. coli* K-12 cells at the top, Figure S4: Fluorescence microscopy analysis of (a) *E. coli*, (b) *L. acidophilus*, and (c) *S. mutans* immunosensing chips that had been incubated with (a) *E. coli* K-12, (b) *L. acidophilus*, and (c) *S. mutan E. coli* K-12 cultures, respectively, at different indicated concentrations (1-10^7^ CFU/10mL), Figure S5: Storage effect on the photocurrent reduction levels of the *L. acidophilus* immunosensing chips on the detection of a 10^4^ CFU/mL *L. acidophilus* culture, Table S1: FTIR-peak (cm^-1^) assignments for pure OA, a mixture of GO and OA (1:5 weight ratio), and pure GO, Table S2: Raman-band (cm^-1^) assignment for the ITO electrodes fabricated with different topmost layers, Table S3: Effects of prior microfluidic washing on the relative averages and relative standard deviations (RSDs) of the total photocurrent densities of the chips fabricated with different topmost layers, Table S4: Comparison of various selective SpA-based immunosensors for microbial detection, Table S5: Sensitivity of *E. coli*, *L. acidophilus*, and *S. mutans* immunosensing chips on the detection of pure *E. coli* K-12, *L. acidophilus*, and *S. mutans* cultures, respectively, in different ranges of cell concentration, Table S6: Ratios of the peak photocurrent values between the light-on and light-off responses of the chips fabricated with different topmost layers for the detection of different cells.
