# Supplementary material for: Versatile Protein-A Coated Photoelectric Immunosensors with a Purple-Membrane Monolayer Transducer Fabricated by Affinity-Immobilization on a Graphene-Oxide Complexed Linker and by Shear Flow
Source: Sensors (Basel). 2018 Dec 18;18(12):4493. doi: 10.3390/s18124493 (PMC6308460; doi:10.3390/s18124493)
Supplement: Supplementary file 1 [file sensors-18-04493-s001.pdf]

Supplementary Materials

Versatile Protein-A Coated Photoelectric Immunosensors with a Purple-Membrane Monolayer Transducer Fabricated by Affinity-Immobilization on a Graphene-Oxide Complexed Linker and by Shear Flow

Hsueh-Hsia Wu [1], Xin-Quan Liao [2], Xin-Ying Wu [2], Cheng-De Lin [2], Kai-Ru Jheng [2], Hong-Ren Chen [2], Yong-Yi Wang [2] and Hsiu-Mei Chen [2,*]

1 School of Medical Laboratory Science and Biotechnology, College of Medical Science and Technology, Taipei Medical University, Taipei 11031, Taiwan
2 Department of Chemical Engineering, National Taiwan University of Science and Technology, Taipei 10607, Taiwan
* Correspondence: hsiumei@mail.ntust.edu.tw (H.M Chen); Tel.: +886-2-2737-6651

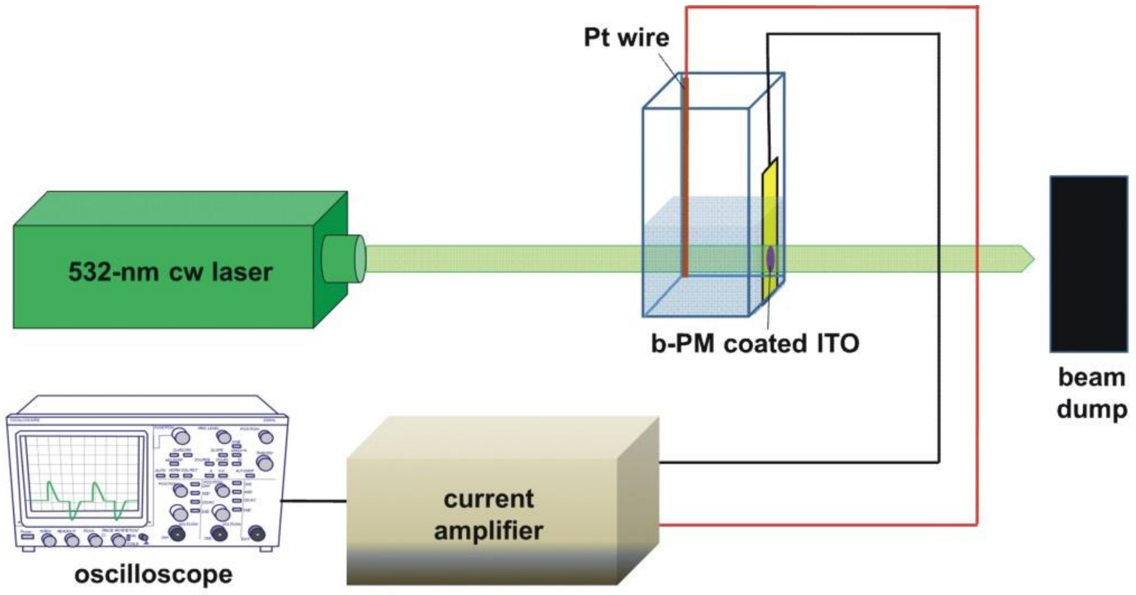

Figure S1. Equipment setup for photocurrent measurement.

Table S1. FTIR-peak (cm^{-1}) assignments for pure OA, a mixture of GO and OA (1:5 weight ratio), and pure GO[a].

OA	Mixture of GO and OA	GO	Band assignment	Reference
	1076	1078	C-O stretching, alkoxy	[S1]
		1265	C-O stretching, epoxy	[S1]
		1358	C-O stretching, COOH	[S1]
1398	1396		C-N stretching	[S2]
1525	1537		Amide II N-H vibrating	[S3-S5]
1639	1635		Amide I C=O stretching	[S3-S5]
		1657	C-C=C symmetric stretching	[S1]
		1714	C=O stretching	[S1]
3289	3292	3375	Hydrogen-bonded O-H stretching	[S1,S6]

[a] Data from the FTIR spectra shown in Figure 2.

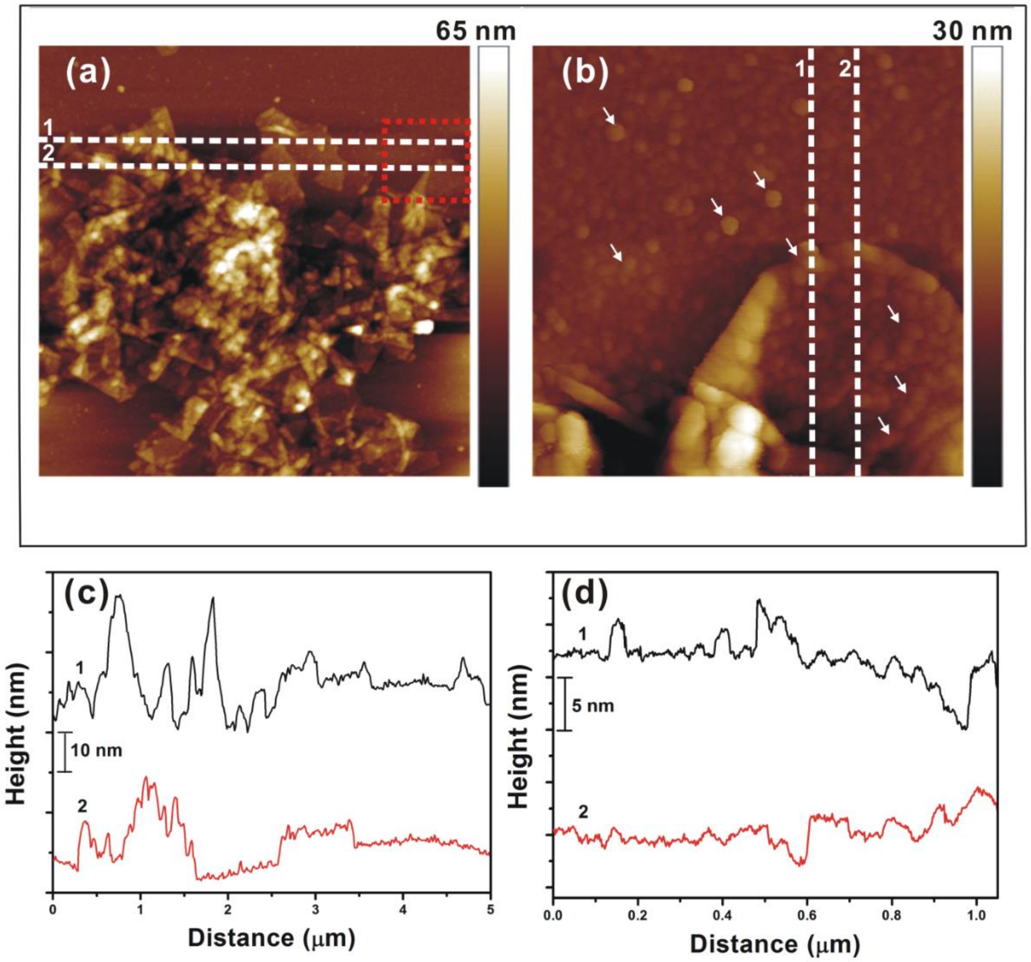

Figure S2. AFM (a, b) topographic images and (c, d) sectional profiles of a GO-OA complex linker deposited on aminated mica. Sectional profiles were analyzed along the white dotted lines on the topographic images. To prepare the AFM sample, GO was mixed with an excess weight amount of OA. After 2.5-h incubation at 4 °C, the mixture was drop-coated on APPA-coated mica for 2 h, washed with deionized water, and then subjected to AFM analysis. Scan size: (a, c) 5 μm and (b, d) 1.1 μm. The red dotted box in (a) and the arrows in (b) indicate the sectioned area for the 1.1-μm scan and OA granules, respectively.

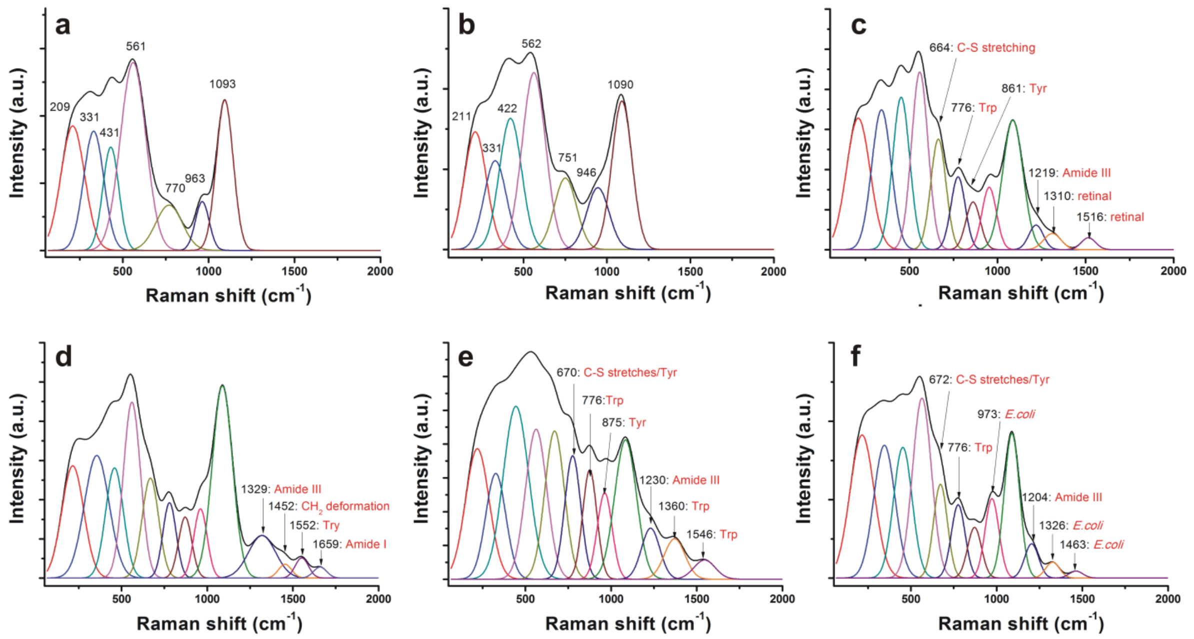

Figure S3. Deconvoluted Raman spectra of ITO electrodes fabricated with (a) APPA, (b) GO-OA complex liker, (c) b-PM, (d) SpA, (e) anti-*E. coli* antibodies, and (f) *E. coli* K-12 cells at the top. The b-PM surface prepared via the complex linker and subsequently washed with a microfluidic shear flow was used for the analysis as well as in the following coatings. Bis(NHS)PEG2 was used for SpA conjugation. A PeakFit deonvolution program was used to identify the bands in each spectrum.

Table S2. Raman-band (cm^{-1}) assignment for the ITO electrodes fabricated with different topmost layers[a].

APPA	GO-OA	b-PM	SpA	Anti-*E. coli* antibody	*E. coli*	Band assignment	References
209	211	209	215	219	215	ITO	-
331	331	341	355	327	345	ITO	-
431	422	453	459	445	453	ITO	-
561	562	559	560	563	565	ITO	-
		664	668	670	672	C-S stretches/Tyr	[S7-S8]
770	751					ITO	-
		776	779	776	776	Trp	[S7-S9]
		861	871	875	871	Tyr	[S7-S9]
963	946	952	961	963	973	ITO/*E. coli*	-/[S10]
1093	1090	1087	1089	1085	1089	ITO	-
		1219		1231	1204	Amide III	[S7,S8,S11]
		1310				C-C-H in-plane bends, retinal	[S12]
					1326	*E. coli*	[S10]
			1329			Amide III	[S11]
				1360		Trp	[S7-S9]
			1454			CH_2 deformation	[S11]
					1463	*E. coli*	[S10]
		1516				C=C stretching, retinal	[S12]
			1552	1546		Trp	[S7,S8,S11]
			1659			Amide I	[S11]

[a] Data from the deconvoluted Raman spectra shown in Figure S3.

Table S3. Effects of prior microfluidic washing on the relative averages and relative standard deviations (RSDs) of the total photocurrent densities of the chips fabricated with different topmost layers[a].

Topmost layer	Without prior microfluidic washing[b]		With prior microfluidic washing[c]	
	Average (%)	RSD (%)	Average (%)	RSD (%)
b-PM	100.00	5.18	100.00	2.99
SpA	40.31	12.80	82.09	2.38
Anti-*E. coli* antibody	39.43	16.53	69.84	1.71
10^4 CFU/mL *E. coli*	39.19	37.27	38.24	6.35
10^6 CFU/mL *E. coli*	36.57	13.76	23.46	3.01

[a] All the data represent the results of three chips of a single kind. The averaged total photocurrent density of the b-PM chips of each kind was used as the standard to calculate the relative averages of the total photocurrent densities of the other chips fabricated with different topmost layers.

[b] b-PM chips without prior washing with a microfluidic flow were analyzed and used for the subsequent coatings.

[c] b-PM chips washed with a microfluidic flow were analyzed and used for the subsequent coatings. Data originated from Figure 6b.

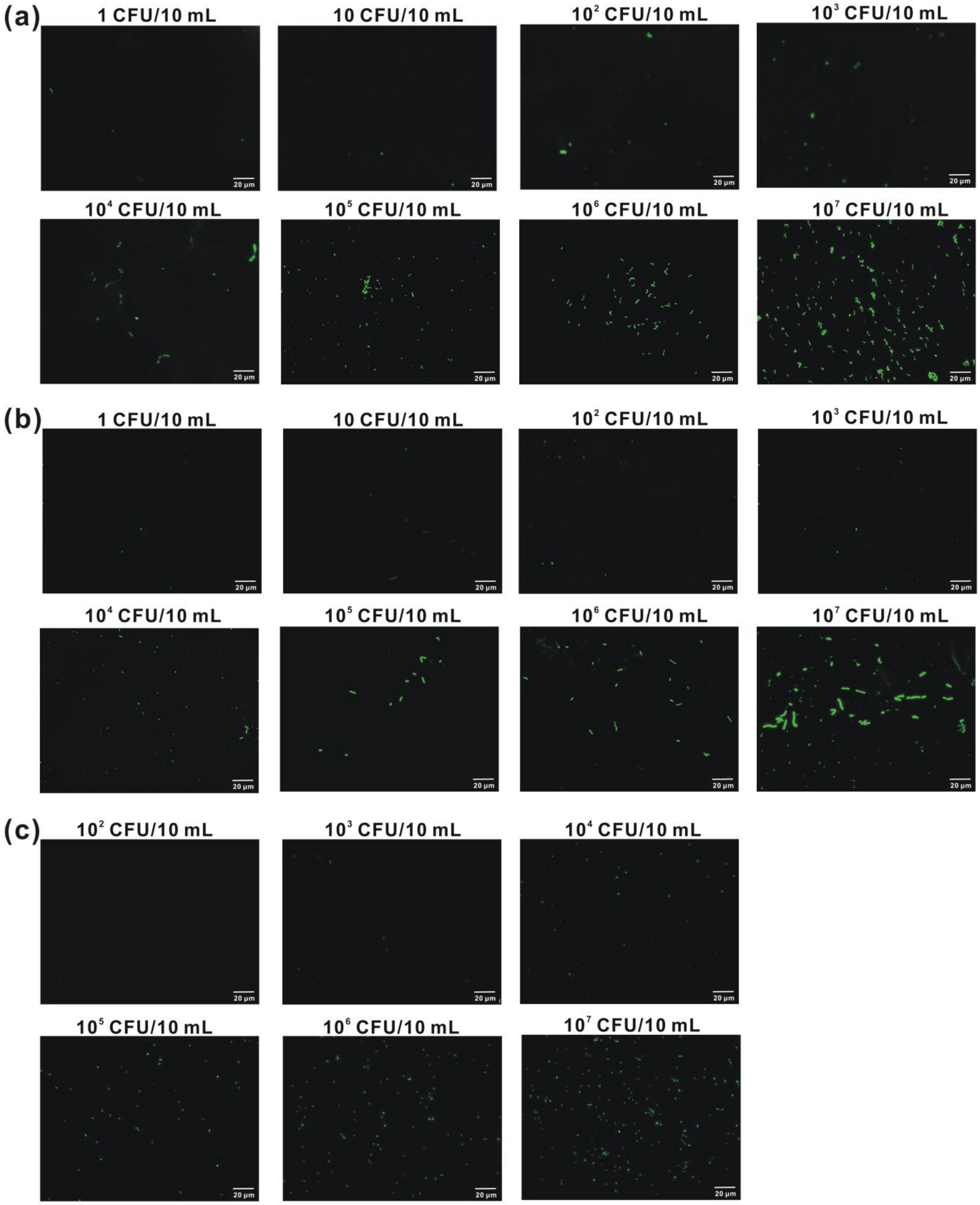

Figure S4. Fluorescence microscopy analysis of (a) *E. coli*, (b) *L. acidophilus*, and (c) *S. mutans* immunosensing chips that had been incubated with (a) *E. coli* K-12, (b) *L. acidophilus*, and (c) *S. mutan E. coli* K-12 cultures, respectively, at different indicated concentrations (1-10^7 CFU/10mL). b-PM chips prepared via the GO-OA complex linker and washed with a microfluidic flow were used to prepare the immunosensing chips. Bis(NHS)PEG2 was used for SpA conjugation. The captured bacteria were stained by SYTO 9 green fluorescent nucleic acid stain and then examined using an Olympus IX73 inverted microscope (Tokyo, Japan). No fluorescent signal was observed on each immunosensing chip incubated just with the blank cell-binding buffer.

Table S4. Comparison of various selective SpA-based immunosensors for microbial detection.

Technique	Direct assay	Label-free assay	Detection limit (CFU/mL)	Dynamic range (CFU/mL)	Assay time	Reference
Bacteriorhodopsin-based photoelectric immunosensor	yes	yes	10^{-1}	10^{-1}-10^{6}	<40 min	This study
Flow bead-injection optical immunosensor	no	no	3×10^{1}	3×10^{3}-4×10^{7}	≈ 17 min	[S13]
Impedance-coupled quartz crystal microbalance immunosensor	no	yes	10^{2}	n.a.[a]	≈ 1 h real-time	[S14]
Nanoparticle-labeled electrochemical immunosensor	no	no	10^{0}	10^{0}-10^{5}	long	[S15]
	no	no	10^{1}	10^{1}-10^{6}	45 min	[S16]
Quartz crystal microbalance immunosensor	yes	yes	10^{3}	7×10^{2}-7×10^{8}	<30 min real-time	[S17]

[a]Not available.

Table S5. Sensitivity of *E. coli*, *L. acidophilus*, and *S. mutans* immunosensing chips on the detection of pure *E. coli* K-12, *L. acidophilus*, and *S. mutans* cultures, respectively, in different ranges of cell concentration[a].

Chip	Cell-concentration range (CFU/mL)			
	**10^{-1}–10^{1}**	**10^{1}–10^{2}**	**10^{2}–10^{5}**	**10^{5}–10^{6}**
E. coli	4.96 ± 0.85[b]	8.34 ± 0.09		14.77
L. acidophilus	8.89 ± 0.35			
S. mutans	n.a.[c]	1.49	9.59 ± 0.32	

[a] Sensitivity was defined as the slope of the calibration curve, i.e., photocurrent reduction level (%)/log (cell concentration (CFU/mL)). Data originated from Figure 7a.

[b] Data represent the mean ± standard deviation of the slope resulting from the linear regression of the mean-value points in each cell-concentration range of the calibration curve.

[c] Not available.

Table S6. Ratios of the peak photocurrent values between the light-on and light-off responses of the chips fabricated with different topmost layers for the detection of different cells[a].

Topmost layer	$\frac{\lvert I_{Peak\text{-}on}\rvert}{\lvert I_{Peak\text{-}off}\rvert}=\frac{R_{p,off}}{R_{p,on}}$		
	E. coli	*L. acidophilus*	*S. mutans*
b-PM	1.26±0.04	1.21±0.03	1.28±0.03
SpA	1.23±0.03	1.16±0.07	1.22±0.02
anti-*E. coli* antibody	1.20±0.03	1.19±0.01	1.25±0.12
Cells (CFU/mL)			
0.1	1.26±0.04	1.07±0.05	–[b]
1	1.15±0.02	1.13±0.02	–
10	1.17±0.02	1.17±0.08	1.27±0.16
10^2	1.20±0.06	1.17±0.08	1.28±0.05
10^3	1.15±0.03	1.08±0.12	1.28±0.08
10^4	1.09±0.06	1.05±0.13	1.17±0.15
10^5	1.05±0.06	1.15±0.07	1.22±0.17
10^6	1.09±0.04	1.12±0.04	1.24±0.10

[a] Data show the averaged ratio of the peak photocurrent values between the light-on and light-off responses of three chips of a single kind with one standard deviation.

[b] Not available.

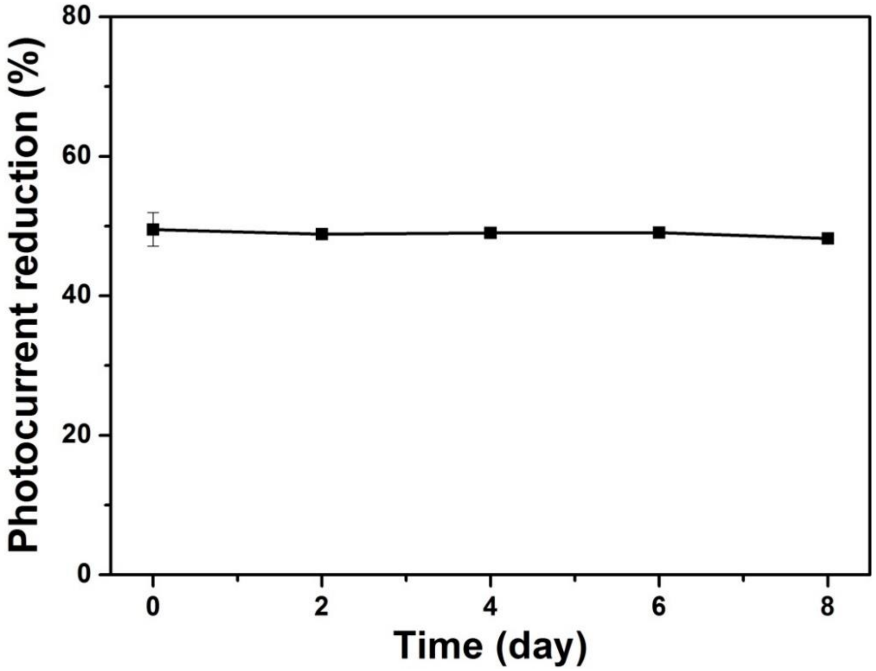

Figure S5. Storage effect on the photocurrent reduction levels of the *L. acidophilus* immunosensing chips on the detection of a 10^4 CFU/mL *L. acidophilus* culture. After the preparation, the antibody-coated chips were either immediately used to detect the culture or stored in a 10 mM phosphate buffer containing 150 mM NaCl (pH 7.4) at 4 °C for 2-8 days. All fresh and the subsequently stored immunosensing chips produced similar total photocurrent densities (1.56±0.04 μA/cm^2). Each datum point represents the average of three chips of a single kind with one standard deviation.

References:

1. Ghosh, T.K.; Gope, S.; Mondal, D.; Bhowmik, B.; Mollick, M.M.R.; Maity, D.; Roy, I.; Sarkar, G.; Sadhukhan, S.; Rana, D.; Chakraborty, M.; Chattopadhyay, D. Assessment of morphology and property of graphene oxide-hydroxypropylmethylcellulose nanocomposite films. *Int. J. Biol. Macromol.* **2014**, *66*, 338–345
2. Yang, Q.; Wang, Z.; Weng, J. Self-assembly of natural tripeptide glutathione triggered by graphene oxide. *Soft Matter*. **2012**, *8*, 9855–9863.
3. Barth, A. Infrared spectroscopy of proteins. *Biochim. Biophys. Acta* **2007**, *1767*, 1073–1101.
4. Young, A.G.; McQuillan, A.J.; Green, D.P. In situ IR spectroscopic studies of the avidin-biotin bioconjugation reaction on CdS particle films, *Langmuir* **2009**, *25*, 7416–7423.
5. Zhao, S.; Walker, D.S.; Reichert, W.M. Cooperativity in the binding of avidin to biotin-lipid-doped Langmuir-Blodgett films. *Langmuir* **1993**, *9*, 3166–3173.
6. Knop, S.; Jansen, T.L.C.; Lindner, J.; Vöhringer, P. On the nature of OH-stretching vibrations in hydrogen-bonded chains: Pump frequency dependent vibrational lifetime. *Phys. Chem. Chem. Phys*. **2011**, *13*, 4641–4650.
7. Painter, P.C.; Koenig, J.L. Raman spectroscopic study of the structure of antibodies. *Biopolymers* **1975**, *14*, 457–468.
8. Rygula, A.; Majzner, K.; Marzec, K.M.; Kaczor, A.; Pilarczyk, M.; Baranska, M. Raman spectroscopy of proteins: a review. *J. Raman Spectrosc.* **2013**, *44*, 1061–1076.
9. Fagnano, C.; Fini, G.; Torreggiani, A. Raman spectroscopic study of the avidin-biotin complex. *J. Raman Spectrosc.* **1995**, *26*, 991–995.
10. Liu, T.T.; Lin, Y.H.; Hung, C.S.; Liu, T.J.; Chen, Y.; Huang, Y.C.; Tsai, T.H.; Wang, H.H.; Wang, D.W.; Wang, J.K.; Wang, Y.L.; Lin, C.H. A high speed detection platform based on surface-enhanced Raman scattering for monitoring antibiotic-induced chemical changes in bacteria cell wall. *PLoS One* **2009**, *4*, e5470.
11. Kengne-Momo, R.; Daniel, P.; Lagarde, F.; Jeyachandran, Y.; Pilard, J.; Durand-Thouand, M.; Thouand, G. Protein interactions investigated by the Raman spectroscopy for biosensor applications. *Int. J. Spectrosc.* **2012**, *2012*, 462901.
12. Braiman, M.; Mathies, R. Resonance Raman spectra of bacteriorhodopsin's primary photoproduct: evidence for a distorted 13-cis retinal chromophore. *Proc. Natl. Acad. Sci. USA* **1982**, *79*, 403–407.
13. Peedel, D.; Rinken, T. Rapid biosensing of *Staphylococcus aureus* bacteria in milk. *Anal. Methods* **2014**, *6*, 2642–2647.
14. Su, X.L.; Li, Y. A QCM immunosensor for *Salmonella* detection with simultaneous measurements of resonant frequency and motional resistance. *Biosen. Bioelectron.* **2005**, *21*, 840–848.
15. Alocilja, E.C.; Jain, P.; Pryg, K. Immunosensor for rapid extraction/detection of enteric pathogens. *Technology* **2016**, *4*, 194–200.
16. Wang, Y.; Alocilja, E.C. Gold nanoparticle-labeled biosensor for rapid and sensitive detection of bacterial pathogens. *J. Biol. Eng.* **2015**, *9*, 16.
17. Hao, R.; Wang, D.; Zhang, X.; Zuo, G.; Wei, H.; Yang, R.; Zhang, Z.; Cheng, Z.; Guo, Y.; Cui, Z.; Zhou, Y. Rapid detection of *Bacillus anthracis* using monoclonal antibody functionalized QCM sensor. *Biosens. Bioelectron.* **2009**, *24*, 1330–1335.
